# How ownership rights over microorganisms affect infectious disease control and innovation: A root-cause analysis of barriers to data sharing as experienced by key stakeholders

**DOI:** 10.1371/journal.pone.0195885

**Published:** 2018-05-02

**Authors:** Carolina dos S. Ribeiro, Martine Y. van Roode, George B. Haringhuizen, Marion P. Koopmans, Eric Claassen, Linda H. M. van de Burgwal

**Affiliations:** 1 Center for infectious Disease Control, The Netherlands National Institute for Public Health and the Environment (RIVM), Bilthoven, Netherlands; 2 Department of Viroscience, Erasmus Medical Center, Rotterdam, Netherlands; 3 Athena Institute for Research on Innovation and Communication in Health and Life Sciences, Vrije Universiteit Amsterdam, Amsterdam, Netherlands; 4 Artemis One Health Research Foundation, Delft, Netherlands; Wadsworth Center, UNITED STATES

## Abstract

**Background:**

Genetic information of pathogens is an essential input for infectious disease control, public health and for research. Efficiency in preventing and responding to global outbreaks relies on timely access to such information. Still, ownership barriers stand in the way of timely sharing of genetic data from pathogens, frustrating efficient public health responses and ultimately the potential use of such resources in innovations. Under a One Health approach, stakeholders, their interests and ownership issues are manifold and need to be investigated. We interviewed key actors from governmental and non-governmental bodies to identify overlapping and conflicting interests, and the overall challenges for sharing pathogen data, to provide essential inputs to the further development of political and practical strategies for improved data sharing practices.

**Methods & findings:**

To identify and prioritize barriers, 52 Key Opinion Leaders were interviewed. A root-cause analysis was performed to identify causal relations between barriers. Finally, barriers were mapped to the innovation cycle reflecting how they affect the range of surveillance, innovation, and sharing activities. Four main barrier categories were found: compliance to regulations, negative consequences, self-interest, and insufficient incentives for compliance. When grouped in sectors (research institutes, public health organizations, supra-national organizations and industry) stakeholders appear to have similar interests, more than when grouped in domains (human, veterinary and food). Considering the innovation process, most of barriers could be mapped to the initial stages of the innovation cycle as sampling and sequencing phases. These are stages of primary importance to outbreak control and public health response. A minority of barriers applied to later stages in the innovation cycle, which are of more importance to product development.

**Conclusion:**

Overall, barriers are complex and entangled, due to the diversity of causal factors and their crosscutting features. Therefore, barriers must be addressed in a comprehensive and integrated manner. Stakeholders have different interests highlighting the diversity in motivations for sharing pathogen data: prioritization of public health, basic research, economic welfare and/or innovative capacity. Broad inter-sectorial discussions should start with the alignment of these interests within sectors. The improved sharing of pathogen data, especially in upstream phases of the innovation process, will generate substantial public health benefits through increased availability of data to inform surveillance systems, as well as to allow the (re-)use of data for the development of medical countermeasures to control infectious diseases.

## Introduction

### The importance for public health

Microbial genetic resources (MGR—here used to indicate strains and genetic sequence data from pathogens and overall microorganisms) are essential inputs to surveillance systems, and therefore of importance to public health. The early identification of changes in pathogens’ genomes are of utmost importance for signaling (re)emerging infectious diseases, developing diagnostic tests and therefore, avoiding possible public health crises. Moreover, the faster these changes occur, the more pressing the need for real time access to pathogens’ strains and genetic data. The changing epidemiology of emerging infectious diseases, with more frequent novel outbreaks and faster spread adds to the fact that efficiency in outbreak control and response relies on timely and global access to MGR [1–3]. The recent Ebola outbreak in West Africa is a clear example, in which gaps in the sharing of viral samples and metadata, and delays in the public release of sequence data led to speculation about sources of infection, ability to diagnose the infection with available assays, and possible changes that increased transmissibility [4, 5]. At the later stages of the outbreak, near real-time sequencing of strains provided information directly to public health officials involved, illustrating its importance [6]. Unfortunately, suboptimal sharing of data occurred throughout the outbreak, allowing the virus to spread and further develop into a global health crisis [7].

### The importance for research and innovation

**Fig 1:**
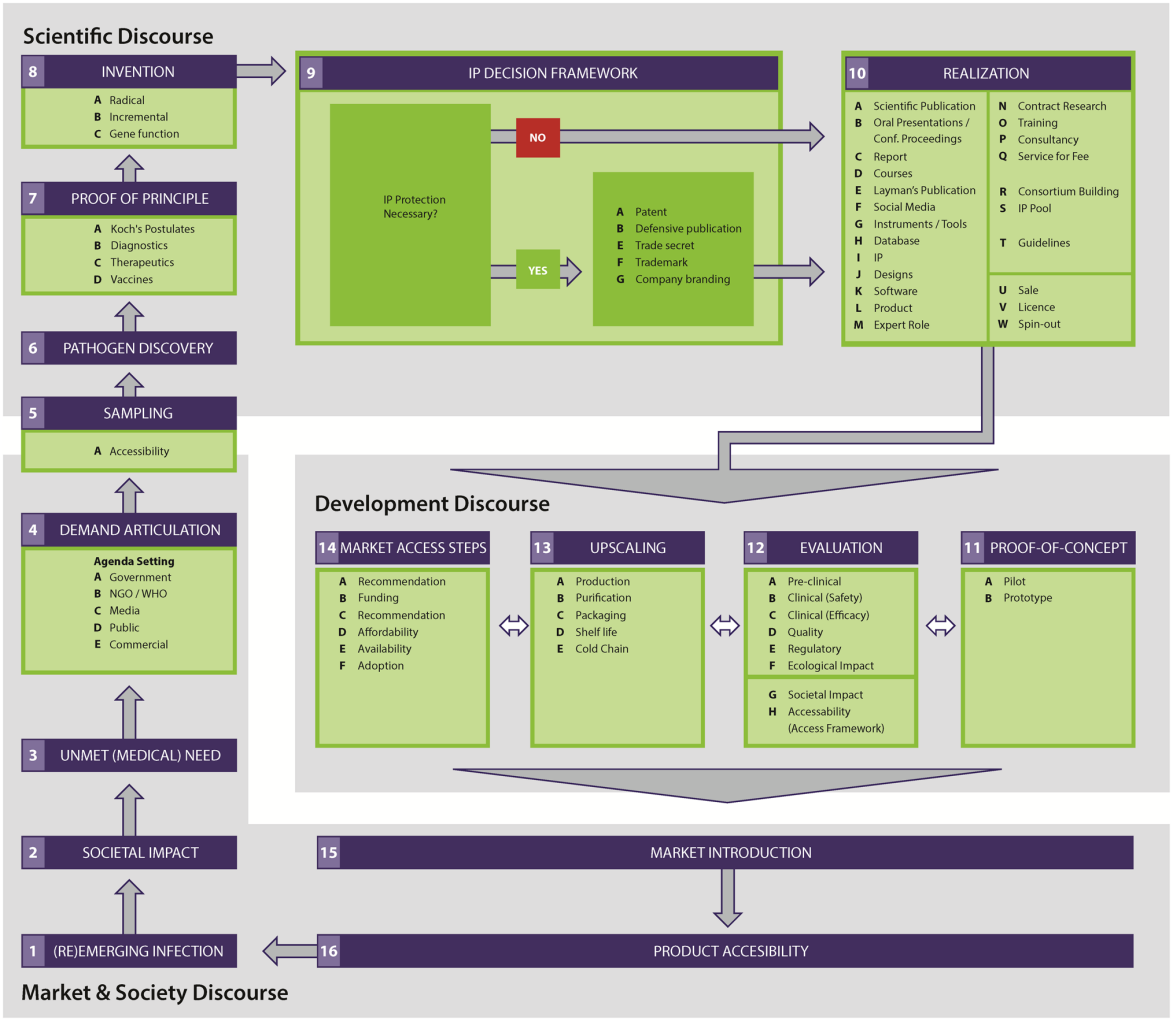
The valorization & technology transfer cycle depicts the steps in the innovation process for infectious diseases control: From disease emergence to development and marketing of medical countermeasures [16]. The upstream phases (steps 1–7) in the cycle overlap with infectious disease surveillance and initial outbreak response, while downstream phases (steps 8–14) overlap with product development as medical countermeasures to control infectious diseases.

Issues preventing and delaying data sharing can be of different nature, and ownership over MGR is certainly an important and complex one. The Nagoya Protocol (NP) [8], for example, was developed to facilitate access to genetic resources and the fair and equitable sharing of benefits arising from their utilization. Nevertheless, despite the importance of reinforcing sovereignty rights of States over genetic resources in their territory, it is hampering the timely access to, and the multilateral sharing of, MGR. By determining that ownership of genetic resources belongs to the government of countries from where the resources originated, the sharing of MGR became a time-consuming bureaucratic process based on bilateral negotiations for access and use [9–11]. Beyond that, the real or perceived possibilities for the commercial valorization of MGR has enforced their appropriation for further use in research, innovation and product development [9]. The problem for public health surveillance occurs when such appropriation is triggered at initial (upstream) phases of the research and innovation cycle, such as sampling and sequencing of microorganisms, instead of later stages, such as the actual product development (in this case drugs, diagnostics and vaccines) [12] (see Fig 1). As such, stakeholders are reluctant to share their (intangible) assets even in early phases of the innovation process [9, 13–16], decreasing the scope of innovation efforts due to the lack of access to upstream research inputs [13, 14, 17]. On the account of this heterogeneous assignment of ownership rights to different actors (private and governmental) at different stages of MGR use, delays in the sharing of and access to MGR has hampered innovations for infectious disease control and ultimately efficient identification, containment and mitigation of outbreaks, with large impacts on public health.

### The multi-stakeholder One Health aspect of MGR sharing

Ownership over MGR is usually perceived as a “bundle of rights”, since rights can be simultaneously allocated to different stakeholders in different situations (e.g. rights to use, modify, share and profit from the resources) [18]. Furthermore, it is increasingly recognized that in order to prevent and control outbreaks of emerging infectious diseases, interdisciplinary collaborations in all aspects of health care for people, animals and the environment are necessary, a phenomenon which is called the One Health approach [19]. Hence, under a One Health approach, the stakeholders involved in the process of producing, sharing and using MGR, and in regulating these activities are diverse, coming from different domains such as human, animal and food. Besides that, relevant stakeholders and their interests are also stratified to different sectors such as public health national institutes, research institutes, supranational organizations and industry. As a result, it is difficult to determine who has the right of ownership of MGR and therefore decides on their sharing [20]. In practice, ownership over MGR is governed by multiple national and international regulations [8, 21, 22], but also determined in private contracts [23, 24]. In addition, regional and international regulations governing data sharing are applicable [25, 26]. In the global health context, national governments decide on the rules for sharing official population data, such as epidemiological and genetic sequence information. Individual scientists, usually the ones producing and sharing this information with either public institutes, universities or the private sector, have to ensure compliance to such rules. On top of that, they must comply with an additional range of agreements and contractual obligations to their employers and research teams. Hence, ownership is an important aspect of MGR sharing, but the allocation of rights is not clear-cut.

### Ownership rights over MGR: Complex and unclear

Ownership rights are not restricted to the decision of sharing and using MGR (property rights), but include a range of other related issues. Based on widely recognized regulations and ethical principles, allocation and transfer of ownership rights are bound to matters of privacy (untraceable data) [3], proportionality (positive risk/benefit ratios) [9, 16], reciprocity (benefit-sharing policies) [8, 9] and consequently responsible use [3, 9] to support societal benefits. Nevertheless, little evidence is available for the in-depth understanding of ownership over MGR and the range of related issues [27]. The technical developments of Next Generation Sequencing technology are being implemented in an extremely rapid pace. The non-technical aspects of implementing this technology, however, are still being broadly discussed between the different stakeholders involved. Such non-technical issues are usually presented in the literature under the PEEARL (political, ethical, economic, administrative, regulatory and legal) framework, but still, such classification scheme fails to provide a clear overview of how ownership issues are perceived by the different stakeholder groups in practice [3, 27].

### The removal of barriers: In search for a constructive approach

In order to establish efficient sharing systems, the engagement and support from the different stakeholder groups are essential. Yet, such wide engagement will not be sustained unless stakeholders’ interests are attained and pondered against possible concerns. This paper investigates such interests and concerns in the view of ownership barriers to the sharing of MGR. It aims to provide an in-depth understanding of how these barriers are perceived by different stakeholder groups in their system of practice. Moreover, we set out to map such barriers in the innovation cycle to identify how they affect surveillance, public health and innovation and understand which phases of the innovation process have a higher sensitivity to barriers hindering the sharing of MGR by specific stakeholder groups. This contributes to the identification of overlapping and conflicting interests of relevant stakeholders, which can be elaborated on constructive discussions on best practices to data sharing, by considering legitimate interests and possibilities for collaboration. Therefore, the ultimate aim of this study is to provide essential inputs to nurture the development of political and practical strategies for improved data sharing practices in an interdisciplinary, international, One Health approach.

## Materials and methods

This study takes qualitative [28, 29] and semi-quantitative approaches to identify and prioritize ownership barriers for the sharing of MGR and to understand in-depth how these barriers influence sharing in the system of practice. To this purpose, interviews were performed with key experts in the field and a root-cause analysis was conducted to analyze the interrelations of barriers and their impact on innovation processes and infectious disease control [30].

### Semi-structured interviews

Semi-structured interviews were conducted with Key Opinion Leaders (KOLs) in the field. Key Opinion Leaders were defined as individuals working in the field of infectious diseases, experienced with the production, sharing and/or use of MGR, and the regulation of these activities, being part of existing networks on this topic. The selection of KOLs was based on an a priori defined population, representative of the MGR sharing environment and identified through the broad network of the EU H2020 funded project COMPARE (http://www.compare-europe.eu). For the sake of representativeness in the study population, the snowballing technique was used to recruit additional study subjects from among respondents’ network [31]. The study population included mainly representatives of organizations from Europe, with the addition of representatives from the United States and China, by involving countries where Next Generation Sequencing technology is being broadly implemented. In accordance to the Dutch law (WMO) [32], the approval from an Ethical Committee was not deemed necessary, since a) this study was not medical in nature and, b) participants were not subject to any actions and/or rules of conduct. We only interviewed key informants about the areas of their competence, therefore the KOLs, as a person, were not the object of the research. Data collection and analysis were performed according to the Chatham House rule of privacy and anonymity [33].

The KOLs were first contacted through email and referred to the EU-COMPARE website for more information about the study design and aim (see the Model of email used to contact participants in S1 File and the Description of the research on the COMPARE website in S2 File). During the telephone interviews, oral informed consent was obtained from all participants, and permission was asked and obtained to record the interviews and to use the anonymized data in further publications. Active informed consent is registered in the interviews’ recordings and transcripts. A standardized topic list was used in the interviews to assess: 1) barriers experienced in their own practice; 2) how the barriers hampered the sharing of MGR; and 3) confirmation if all perceived barriers were mentioned and/or if respondents had any additional comment (see the Interview guide in S3 File). By delineating these three categories in each interview, the interpretative frame of the KOLs could be identified, such as how the barriers related to the context of the respondents, the perceived causes and consequences of the barriers within this context, and the respondents’ main concerns [34]. After identifying the interpretative frames, they were compared and finally integrated in the data analysis. During the interviews, the KOLs were asked to identify barriers in relation to microbial genetic data, which was the initial focus of this study. Nevertheless, as microbial physical samples (strains) and genetic sequence data are inextricably linked in a continuous process of production, the KOLs mentioned barriers that relate to both resources. When KOLs were asked to explain how the mentioned barriers hamper the sharing of MGR, however, it was made clear whether they were referring to microbial samples, data or both. This distinction was used in subsequent steps of data analysis to identify which step of the innovation cycle was hampered by specific barriers.

### Data analysis

The interviews were recorded, transcribed, and coded to ensure anonymity of the KOLs. Interviews were thematically analyzed according to an a priori established coding framework that was complemented during the analysis by the emergence of new themes and codes (see the Coding guide in S1 Dataset). Five predetermined steps were followed: familiarization, identifying the thematic framework, data indexing, data charting, data mapping, and data interpretation; consistent with the standard practice in qualitative data research [35, 36]. Subsequently, two researchers (CdSR and MvR) analyzed the data separately by identifying barriers and their causal factors. Initial analyses were discussed in the team for the construction of the causal trees. Next, barriers were quantified by their frequency of occurrence, as mentioned by the KOLs, revealing their relative importance and whether they were broadly recognized or specific to certain stakeholder groups. When barriers were equally mentioned the following criteria were used for prioritization: 1) their occurrence as mentioned by multiple stakeholder sectors, with barriers mentioned by all sectors receiving a higher ranking, and 2) their occurrence as mentioned by the national surveillance centers as national authorities, which inherit the ultimate decision-making power from local governments in national jurisdictions.

### Mapping to the innovation cycle

Finally, the barriers were plotted on the valorization and tech transfer cycle (Fig 1) to identify how they impact the full range of surveillance, innovation, and sharing activities for infectious disease response and control [28, 29]. This was performed by the first authors (CdSR and MvR), through the distinction of processes (steps) within the cycle that were potentially hampered by each barrier, based on the interpretative frame of the KOLs and the identification of causal factors. This definition was subsequently discussed within the research team for the final analysis.

## Results

**Fig 2:**
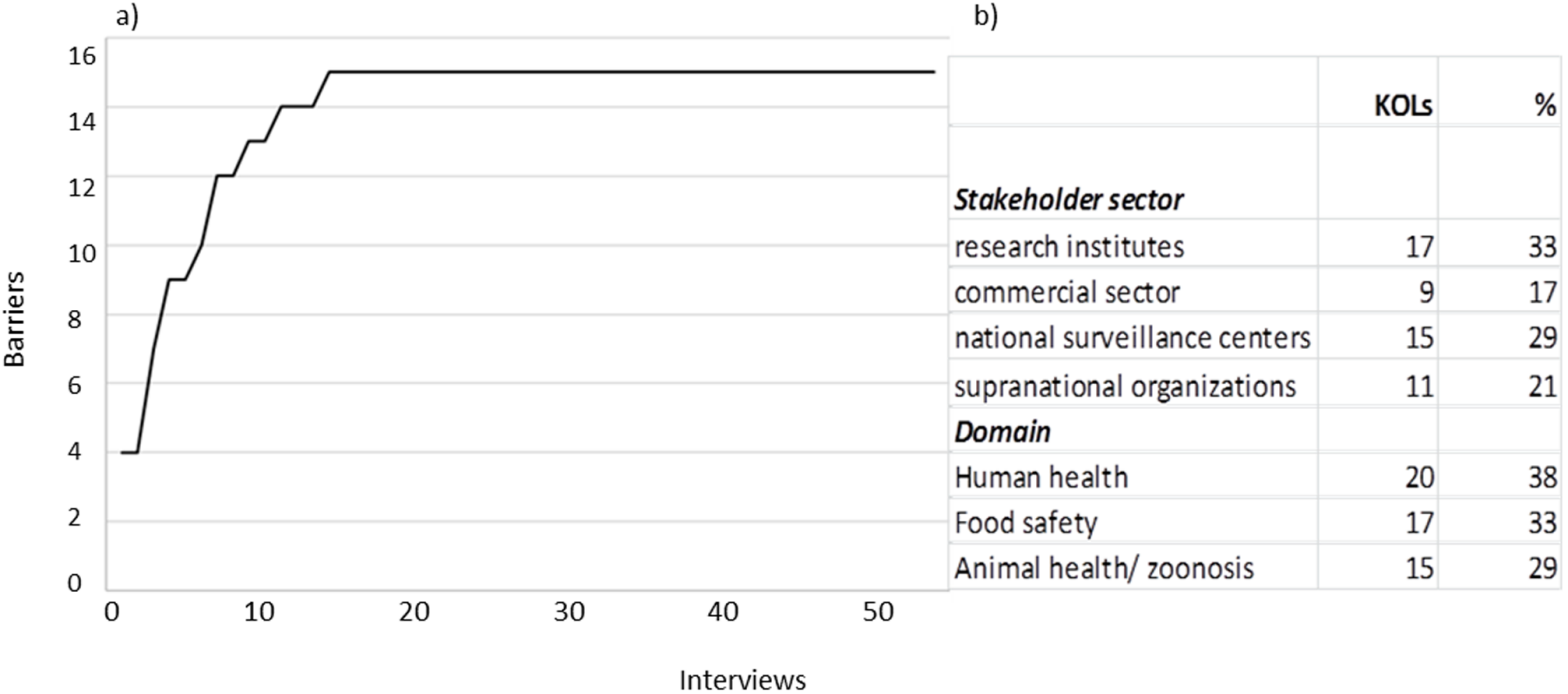
Saturation of ownership barriers for MGR sharing was reached after 14 interviews (a). Representation of KOLs across stakeholder sectors and domains (b). The choice in stakeholders’ sectors and domains reflects their main area of expertise, since the KOLs can be active across two or more domains and sectors.

In total, 52 KOLs were interviewed, primarily working in research institutes (n = 17) the commercial sector (n = 9), national surveillance centers (n = 15), and supranational organizations (n = 11). The KOLs represented the human health (n = 20), food safety (n = 17), and animal health/ zoonosis (n = 15) domains (Fig 2b). Saturation of the mentioned barriers was achieved after 14 interviews (Fig 2a). Data collection continued after reaching the saturation point, as the selection of KOLs was based on a population representative of the MGR sharing environment, and continuing the interviews was deemed justified based on the right of inclusion as well as for the sake of representation.

The root-cause analysis defined 15 unique barriers that currently hamper timely sharing of and open-access to MGR. These barriers were classified in four categories: compliance to regulations before data can be obtained and shared (section A); negative consequences of data sharing for data owners (section B); self-interest of data owner to profit from the data before sharing (section C); and insufficient incentives for compliance to polices supporting timely and openly data sharing (section D).

### A) Compliance to regulations (Fig 3)

**Fig 3:**
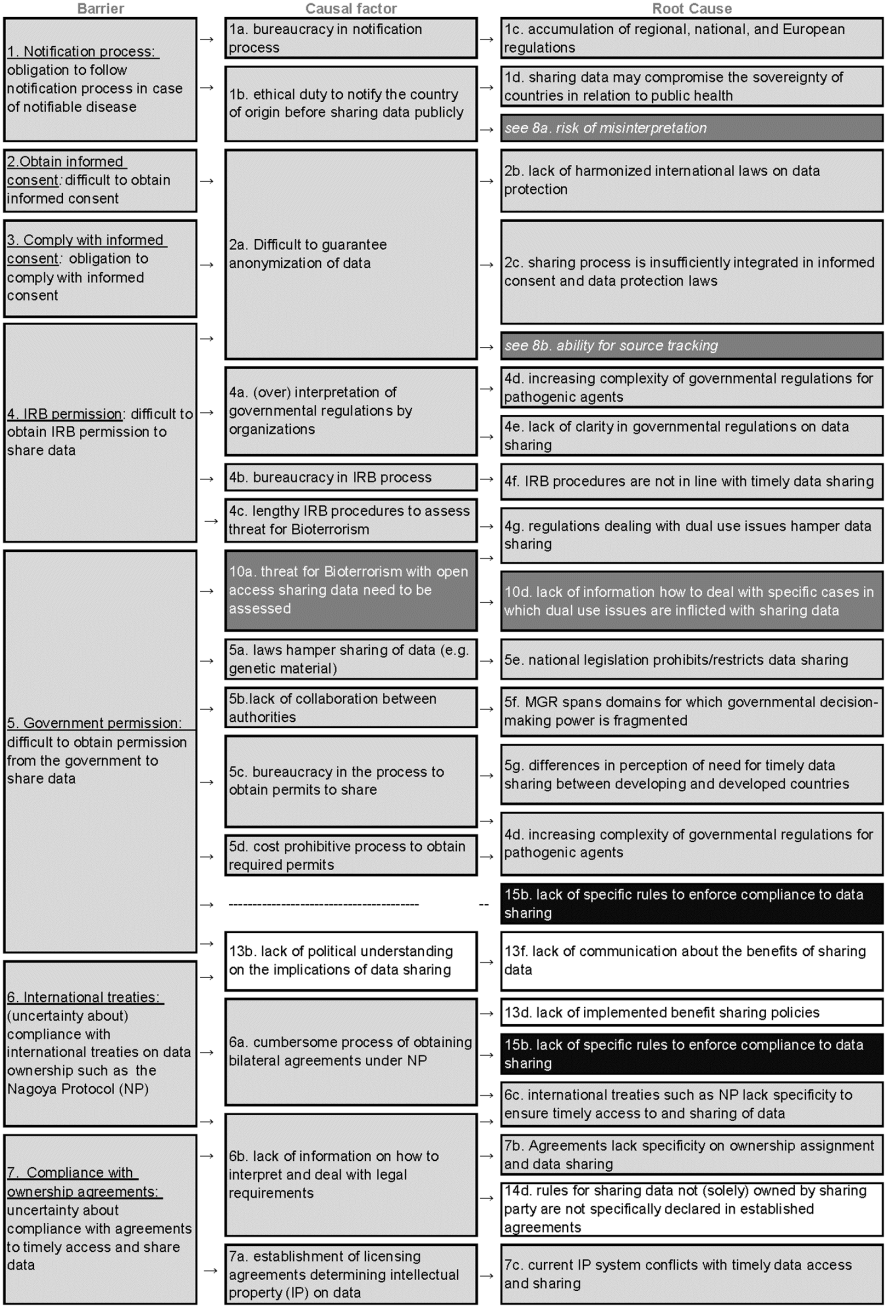
Root-cause analysis of the seven barriers classified under the category “compliance to regulations” (A). Columns represent barriers, causes and root causes, and rows represent the causal argumentation of the KOLs. The barriers are shaded and numbered following the thematic classification in barriers types as the following: compliance to regulations (A, #*1–7* are light grey), negative consequences (B, #8–10 are dark grey), self-interest (C, #11–14 are white) and insufficient incentives for compliance (D, #15 is black).

Seven unique barriers related to this barrier category. Overall, the causal tree revealed 21 different root-causes for the difficulties in compliance with regulations. Most root-causes in this tree related to the lack of standardization and specificity in regulations, added to unclear and restrictive policies. Several root causes were common to more than one barrier category.

### B) Negative consequences (Fig 4)

**Fig 4:**
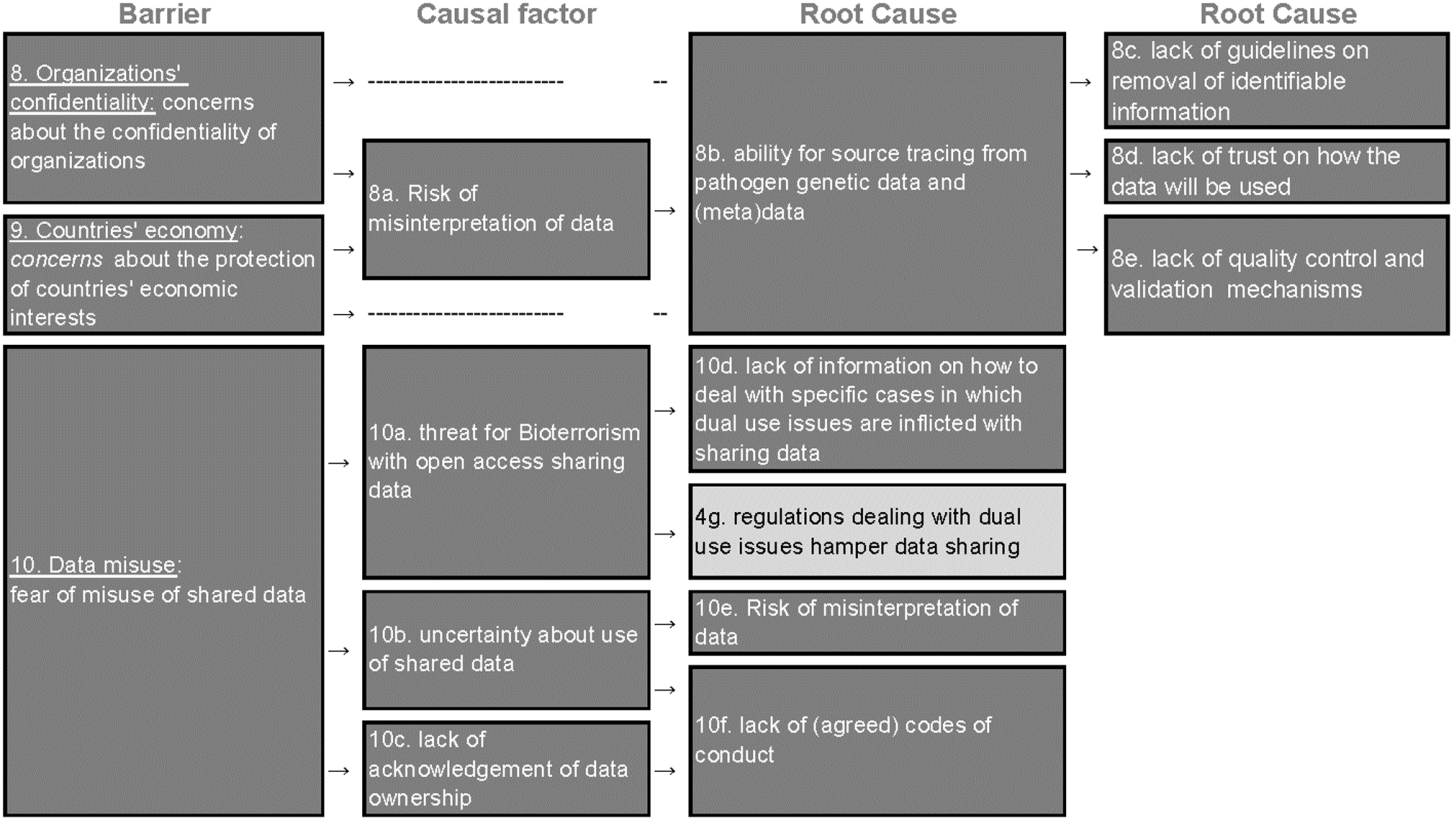
Root-cause analysis of the three barriers classified under the category “negative consequences” (B). Columns represent barriers, causes and root causes and rows represent the causal argumentation of the KOLs. The barriers are color-coded and numbered following the thematic classification in barriers types as the following: compliance to regulations (A, #1–7 are light grey), negative consequences (B, #8–10 are dark grey), self-interest (C, #11–14 are white) and insufficient incentives for compliance (D, #15 is black).

The second barrier category is composed of three unique barriers reflecting concerns about the confidentiality of organizations; protection of countries’ economic interests; and fear of data misuse. In total, seven root-causes and four causal factors were identified. Most of the root-causes underlying these barriers related to uncertainty and the lack of control over the use of the data once it is shared.

### C) Self-interest (Fig 5)

**Fig 5:**
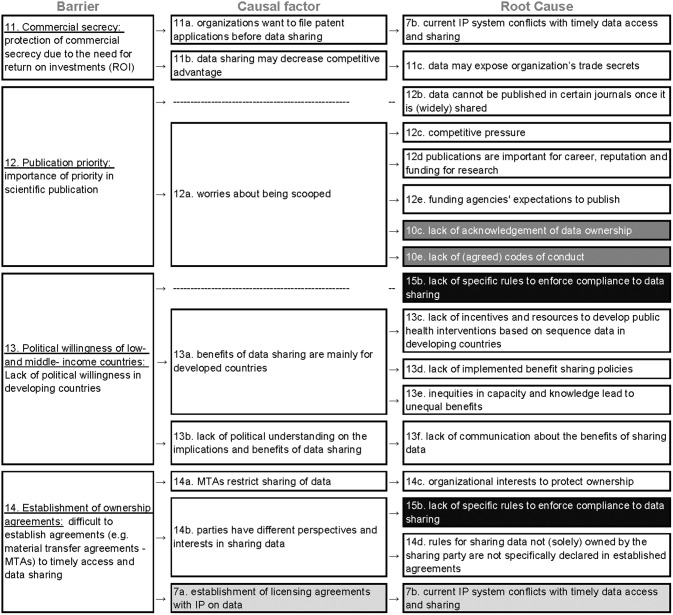
Root-cause analysis of the four barriers classified under the category “self-interest” (C). Columns represent barriers, causes and root causes and rows represent the causal argumentation of the KOLs. The barriers are color-coded and numbered following the thematic classification in barriers types as the following: compliance to regulations (A, #1–7 are light grey), negative consequences (B, #8–10 are dark grey), self-interest (C, #11–14 are white) and insufficient incentives for compliance (D, #15 is black).

Four unique barriers were related to self-interest from MGR owners to profit from the data before sharing it. Overall, 16 root-causes were revealed, four of which originated from other barrier types (A, B, and D).

### D) Insufficient incentives for compliance (Fig 6)

**Fig 6:**
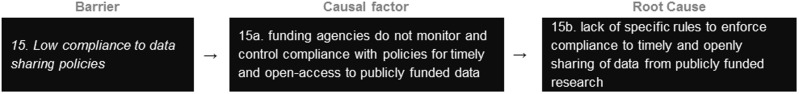
Root-cause analysis of the barrier classified under the category “insufficient incentives for compliance” (D). Columns represent barriers, causes and root causes and rows representing the causal argumentation of the KOLs. The barriers are color-coded and numbered following the thematic classification in barriers types as the following: compliance to regulations (A, #1–7 are light grey), negative consequences (B, #8–10 are dark grey), self-interest (C, #11–14 are white) and insufficient incentives for compliance (D, #15 is black).

The last category of barriers relates to lack of incentives for compliance with policies for MGR sharing. It constitutes an *à contrario* argument, meaning that the barrier does not refer to a mechanism that is in place, but rather to insufficient or lack of existing mechanisms to properly motivate data sharing. The unique root-cause identified relates to the lack of specification in rules to ensure that data produced in publicly funded research are made available to the public in an open-access and timely manner.

### Barriers mapped to the innovation cycle

**Fig 7:**
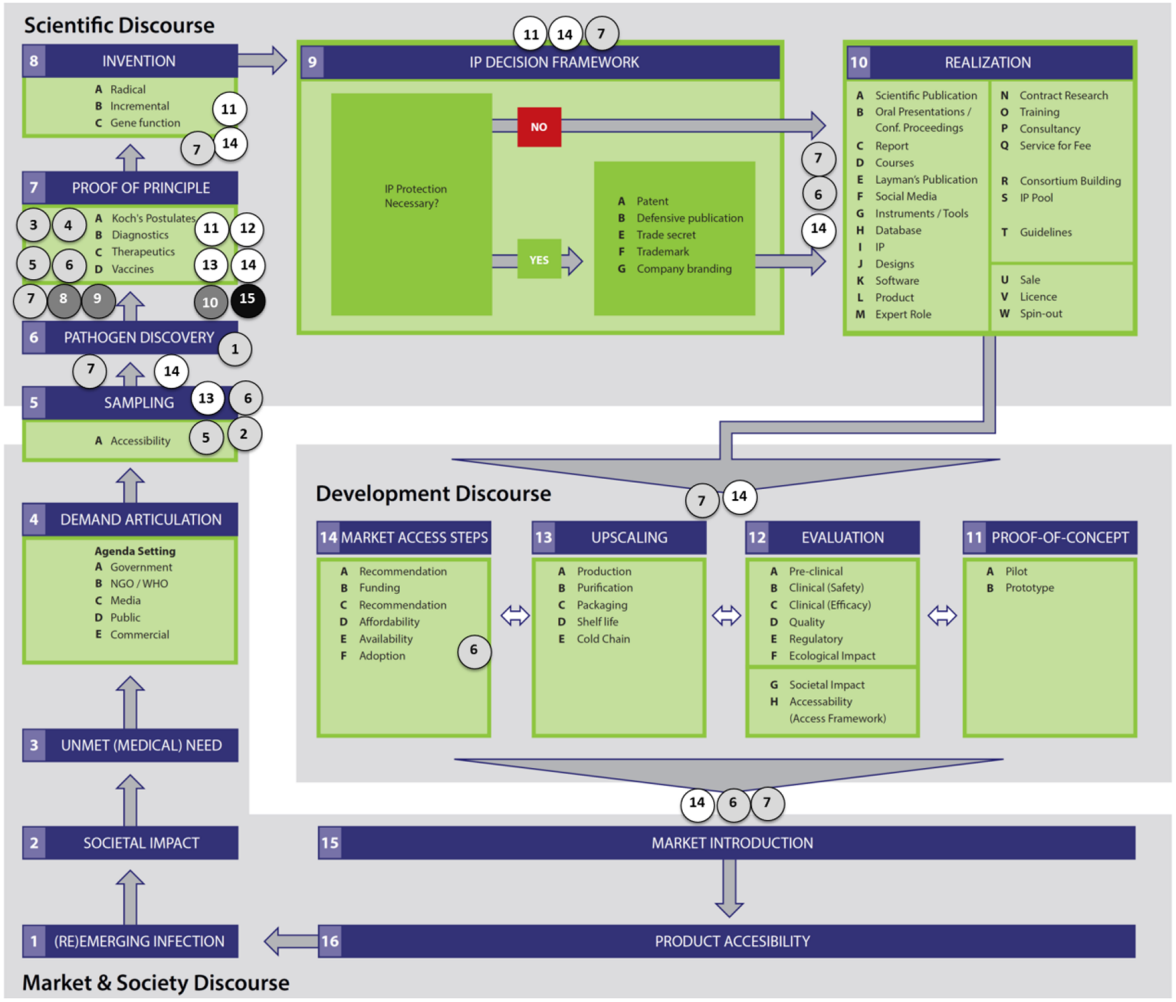
The barriers predominantly influence the scientific domain and the transition between the market & society to the scientific domain. Color codes and numbering correspond to the main barriers and underlying causes in the root-cause analysis trees, in Figs 3–6: A) compliance to regulations (light grey), B) negative consequences (dark grey), C) self-interest (white), and D) insufficient incentives for compliance (black).

**Fig 8:**
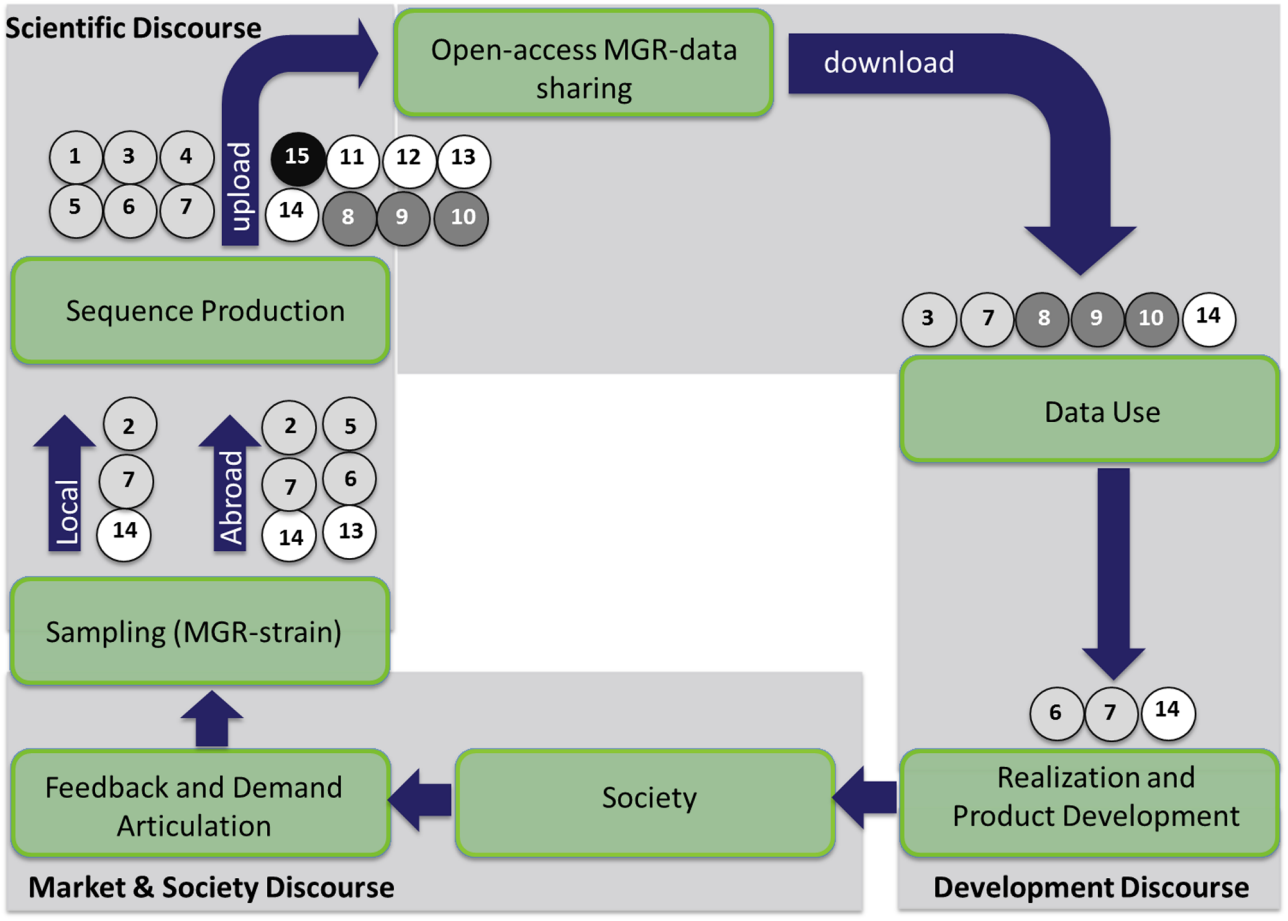
Ownership barriers for timely sharing and open-access to MGR predominantly hamper the innovation process for infectious diseases in the phases from sampling through proof of principle. Color codes and numbering correspond to the main barriers and underlying causes in the root-cause analysis trees, in Figs 3–6: A) compliance to regulations (light grey), B) negative consequences (dark grey), C) self-interest (white), and D) insufficient incentives for compliance (black).

The barriers were plotted in the valorization and technology transfer cycle to illustrate how they influence innovation processes (Fig 7). The barriers affecting the steps 5 (sampling) and 6 (pathogen discovery) in the cycle are related to microbial genetic samples (strains), while barriers from the step 6 onwards relate to microbial genetic data. Although barriers affect all domains of the cycle, the predominant influence was found in the scientific domain and in the transition from market & society to the scientific domain, related to the phases from sampling until proof-of-concept. Furthermore, the figures (Figs 7 and 8) reveal that barriers classified as compliance to regulations (causal tree “A”) and self-interest (causal tree “C”) have a predominant influence on the innovation process by impacting several steps in all domains of the cycle. Mostly, these barriers are related to the (uncertainty about) compliance with international treaties on data ownership (A#6), as the NP, and the difficulty to establish and comply with ownership agreements (A#7 and C#14). Additionally, a crucial differentiation, as revealed in Fig 8, is whether sequencing is performed locally or outside the country of origin of the sample. The exportation of MGR-strains would make access and sharing more difficult since it requires government permission (A#5), in compliance with international treaties (A#6), which depends on government political willingness to share (C#13).

### Quantification of barriers

**Fig 9:**
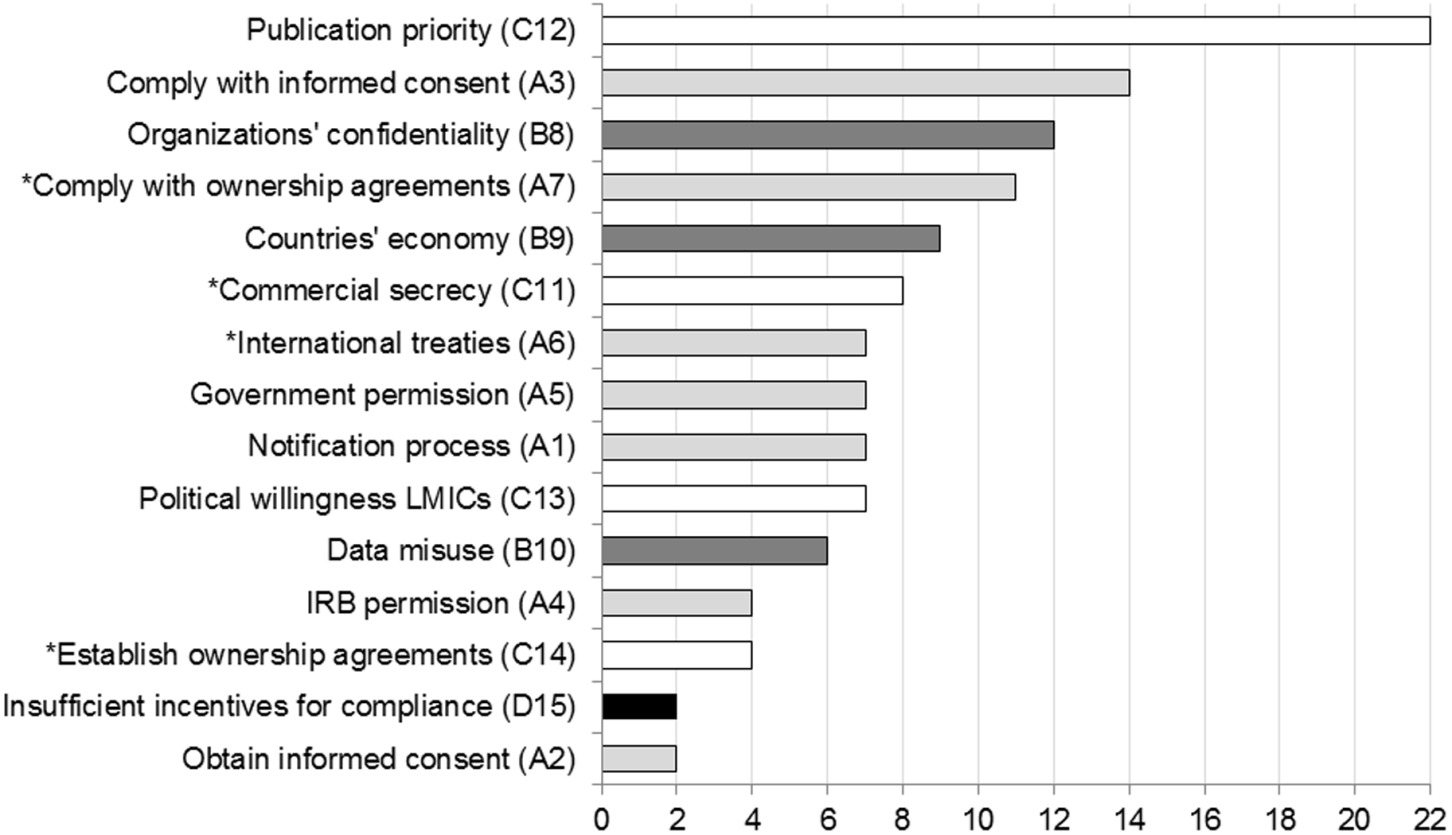
Publication priority was the most frequently mentioned barrier. Barriers are ranked according to their frequency of occurrence in absolute numbers (*n* KOLs) and in decreasing order. Color codes and numbering correspond to the main barriers and underlying causes in the root-cause analysis trees, in Figs 3–6: compliance to regulations (A#x light grey), negative consequences (B#x dark grey), self-interest (C#x white), and insufficient incentives for compliance (D#x black). The asterisk identifies barriers that persist in the downstream phases of the innovation cycle (Fig 7).

The most frequently mentioned barrier was publication priority, mentioned by 22 of the 52 KOLs (Fig 9). The most mentioned barrier category was compliance to regulations (mentioned by 39 of the 52 KOLs, light grey barriers in Fig 9). The second most mentioned category was self-interest (mentioned by 30 KOLs, white bars), including the most mentioned individual barrier. This was followed by negative consequences (23 KOLs, dark grey bars), and insufficient incentives for compliance (2 KOLs, black bar).

**Fig 10:**
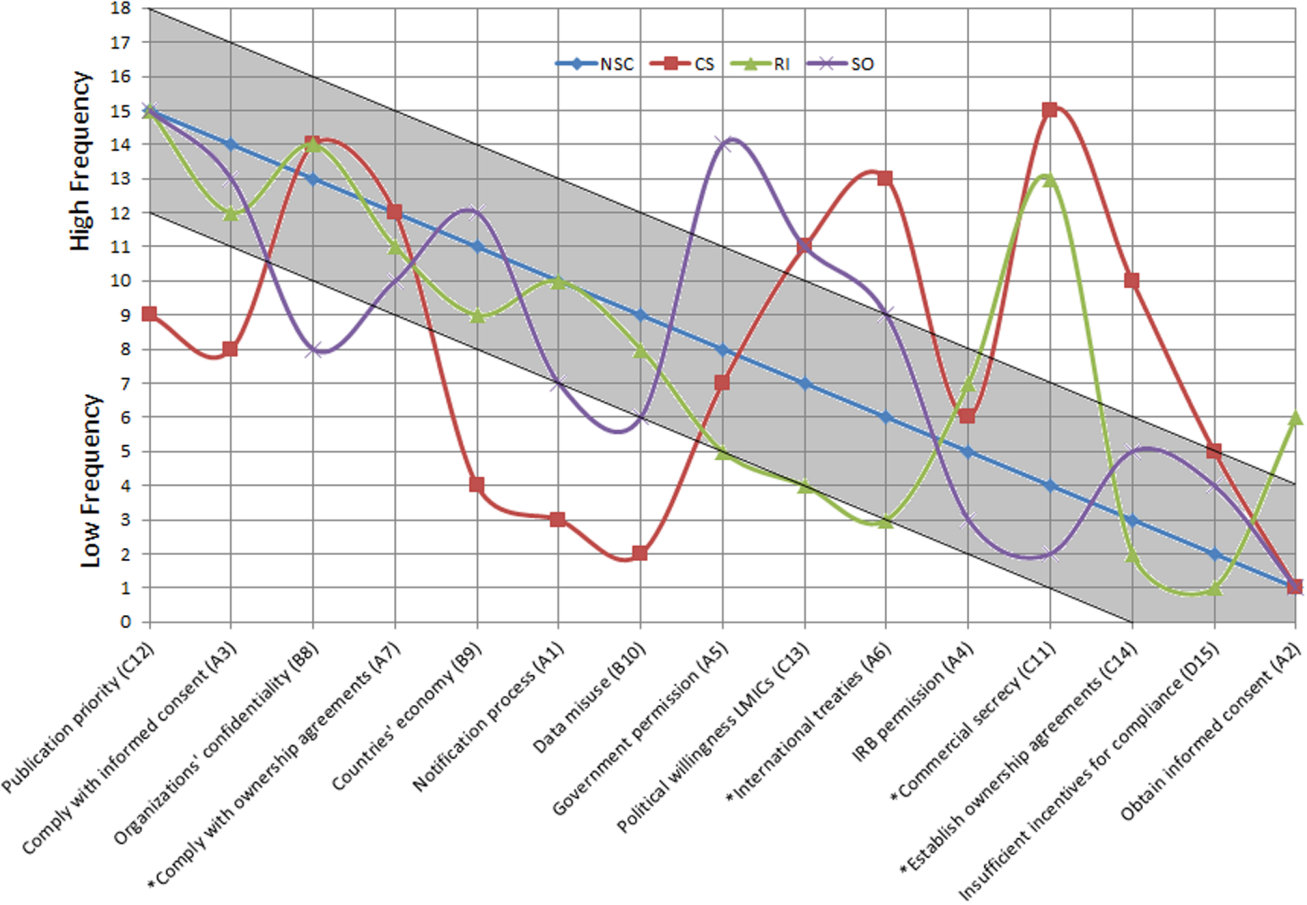
The commercial sector (CS) differentiated substantially from the other stakeholder groups in the quantification of the mentioned barriers. The order generated by the frequency of occurrence as mentioned by the national surveillance centers (NSC) is taken as a reference, to which the behavior of the curves representing the commercial sector (CS), research institutes (RI), and supranational organizations (SO) is compared. Barriers are numbered according to the root-cause analysis (Figs 3–6) and the asterisk identifies barriers that persist in the downstream phases of the innovation cycle (Fig 7).

When the quantification of barriers was stratified per stakeholder sector, it became apparent that the commercial sector differs substantially from the others (Fig 10). Notably, they more frequently mentioned barriers that persist at downstream phases of the innovation cycle such as compliance (A#7) and establishment (C#14) of ownership agreements, international treaties (A#6), and commercial secrecy (C#11). Likewise, a relatively lower frequency in occurrence, for KOLs from the commercial sector, is noted for some barriers at upstream phases such as publication priorities (C#12), compliance with informed consent (A#3), countries economy (B#9), notification process (A#1), and data misuse (B#10). Moreover, although less prominent, research institutes highly mentioned the barrier commercial secrecy (C#11) and obtaining informed consent (A#2). For the supranational organizations, the most mentioned barriers were government permission (A#5) and political willingness of low- and middle- income countries (C#13).

## Discussion

This study identified 15 ownership barriers within four broad categories that critically hamper the timely and openly sharing of MGR. The root-cause analysis revealed that barriers are complex and entangled, due to the diversity of causal factors and their crosscutting features. The four most mentioned barriers were publication priority, compliance with informed consent, organizations’ confidentiality and compliance with ownership agreements. Although priorities differ per stakeholder sector, barriers appeared to affect predominantly early phases of the innovation cycle, confirming their potential impact on the use of MGR for surveillance activities. Some critical barriers that result from sequencing abroad, such as the need of government permission (A#5), international treaties (A#6) and governments political willingness (C#13), can be surpassed by performing sequencing in the country of origin of the strain and/or outbreak. A limited set of barriers seemed to be crucial for downstream commercial applications, as primarily mentioned by KOLs from the commercial sector. The prominent prioritization of barriers in downstream phases of the innovation cycle, by this sector, highlights their primary position as end-users in the sharing process.

### Economic, reputational and personal fears

The most frequently mentioned barriers related to uncertainties implied in the process of sharing MGR, either due to possible financial and reputational losses, or due to exposure of sensitive information. Publication priority, as the most mentioned barrier, revealed the importance of guaranteeing reputational returns to research efforts, as increasingly recognized in the scientific literature [2, 3, 9, 10, 24, 37–39]. Contrary to what might be expected, it is interesting to notice here that the lack of incentives for compliance to data sharing policies (D#15) was not mentioned as often as the barrier of publication priority, while the former can be interpreted as an *à contrario* motivation to the latter. An explanation might be found in the difference of the nature and appearance of both barriers. Publication priority to assure one’s scientific interest is a deliberation experienced by most or all researchers, while the question of strong enough incentives to share is more a consideration that will be present on a managerial level, concerned about compliance to sharing policies. Nevertheless, and speaking of publication priority as a first barrier, the establishment of alternative performance indicators in data sharing policies, like acknowledgment of data sharing contributions (B#10c; C#12d) and the inclusion of such criteria to the assessment of academic research credit, could create trust and incentives for sharing and therefore alleviate this barrier [2, 36]. The second and third most mentioned barriers related to concerns about the protection of sensitive data from individuals, organizations and countries [40]. The potential identification of sensitive data, due to triangulation of sources [4], can infringe on privacy and informed consent policies, generate stigma and, in some cases, may even be used as a legal instrument for prosecution [41]. In addition, source tracing can have negative reputational and economic impacts for organizations and countries through the interruption of commerce and trade. Solutions for these barriers should focus on their causal factors such as the absence of an agreed code of conduct (B#10e), lack of quality control and validation mechanisms (B#8e), and insufficient integration of data sharing in the informed consent process (A#2c). The development of ‘controlled data hubs’, for instance, could be used as a temporary solution where different levels of access—open access and controlled access—could address uncertainties and prevent reluctance to share [4]. Finally, the barrier of compliance with ownership agreements was frequently mentioned. Causal factors for this barrier related to the lack of adequate information on the legal requirements of agreements (A#6b, 7#b) and global legal standards (A#2b), which generate diverse interpretations in the allocation of ownership rights over MGR (A#7b). To address this barrier, standardized ownership agreements in which associated rights and permissions for (re-)use are clearly tagged on the data, need to be established [4]. Examples are the establishment of multilateral systems for global data sharing, with standard material transfer agreements (MTAs) [9], and the use of permissive open source licenses that facilitate data access, use and sharing [42].

### Public health, research & innovation or economic welfare?

Most barriers were encountered in early phases of the innovation cycle, from sampling through proof of principle. Importantly, barriers affecting the sharing of MGR in upstream stages of innovations will reflect on downstream health-related products developments and therefore, ultimately influence their benefits for public health [7, 13–15]. The differences in ranking of barriers between stakeholder groups indicate the need for refinement in the discussion about specific barriers. For instance, the main concern of stakeholders from the private sector relates to the uncertainties and ambiguity in regulatory and governance mechanisms to the sharing of MGR (A#6), as well as to the protection of return on investments (C#11 and #14). This suggests that this sector should not necessarily be involved in the development of solutions for barriers that affect upstream phases of innovations, as long as the solutions lead to clearer mechanisms for (re-)use of MGR and allow for the appropriation of resulting innovations. This perception, however, changes if stakeholders from the commercial sector are also considered potential data producers and contributors, such as food companies, or companies developing diagnostic instruments with surveillance applications. For research institutes, an important concern is also at downstream phases of innovation process, as noted in the high prioritization of the barrier commercial secrecy (C#14). This highlights that although academia have the main concern to generate societal benefits through their research, they usually have to operate on a commercial basis by investing in downstream valorization of research results in order to guarantee sufficient reputational and, in some cases, financial returns for future research [9]. Supranational organizations have as their main concerns to procure government permission (A#5) and countries’ political willingness (C#13) for sharing. A possible reason for this is the mandate of such supranational authorities to defend national interests, which generates dependency on the interests of Member States.

### A way forward: Articulating barriers within stakeholder groups

A consistent causal factor mentioned for all categories of barriers and by all stakeholder sectors was the many (legal) uncertainties that exist in the context of sharing MGR. This indicates the need for simplification, education and clarification of legal frameworks, international treaties, and existing legislation [3, 4]. If rules and obligations for data sharing are known to all stakeholders from the outset and throughout the sharing process, a climate of trust and reciprocity can be established, contributing to a sustainable sharing environment [2]. Consequently, enforcing such a climate through the development of soft law arrangements, like codes of conducts and enforcements of community norms, can help to build consensus on the core values of the system [11]. Even though certain barriers appear to be of more importance to certain sectors, collaboration between stakeholders is necessary, with an active exchange of experiences and best practices [43]. The first step, however, should be sought in creating awareness and consensus within sectors on the causal factors of barriers. As saturation of barriers in the interviews was achieved relatively quickly, the stakeholders share a common view on the overall barriers to the sharing of MGR. Nevertheless, the multitude of causal factors revealed that stakeholders have different interpretations to what factors cause barriers and how they affect the sharing process. The diversity in stakeholders’ motivations for sharing MGR, whether the priority should be the importance for public health, basic research, economic welfare and/or innovative capacity, highlights the fact that interests frequently overlap among stakeholders from the same sector, more than when stakeholders are grouped in domains. This increased identity of stakeholders with peers from the same sector also leads to an accentuated difference of interests between stakeholders from different sectors. On these grounds, sectors should first articulate their main interests before discussing them with other stakeholder groups. In this way, awareness about different perspectives can be raised, common goals established and a focused discussion towards overcoming barriers for timely sharing of and open-access to MGR realized.

## Conclusion

Obviously, advances in molecular technology, openness and sharing of information on the digital arena will not slow down. Hence, we need to be prepared to adopt them consciously, without infringing the interests of the public and other stakeholders. By identifying barriers and considering them according to the perspective and underlying interests of key stakeholders, we could identify overlapping and conflicting issues that occur in alignment with the grouping of stakeholders in sectors. The investigation and understanding of such interests within and between stakeholder sectors are essential to the development of collaborations and solutions to accelerate the sharing of MGR in an ethical, legal and efficient manner. This efficiency in the sharing of MGR, especially in upstream phases of the innovation process, will generate substantial public health benefits through increased availability of data to inform surveillance systems, as well as through allowing the (re-)use of data for the development of medical countermeasures to control infectious diseases.

## Supporting information

S1 File Model of email used to contact participants. (PDF)

S2 File Description of the research on the COMPARE website. (PDF)

S3 File Interview guide. (PDF)

S1 Dataset Coding guide. Description of the codes that emerged from the interviews and the definition of the final coding scheme, including illustrative quotes.
(XLSX)
