# Supplementary material for: How ownership rights over microorganisms affect infectious disease control and innovation: A root-cause analysis of barriers to data sharing as experienced by key stakeholders
Source: PLoS One. 2018 May 2;13(5):e0195885. doi: 10.1371/journal.pone.0195885 (PMC5931471; doi:10.1371/journal.pone.0195885)
Supplement: S1 File — (PDF) [file pone.0195885.s001.pdf]

S1 File. Model of e-mail used to contact participants.

Dear [title] [name],

In the EU COMPARE project a specific work package is dedicated to study barriers for timely and openly sharing of microbial genetic data in a publicly accessible international database. Following the survey on sharing real-time/rapid open source metadata we performed in January of this year, we are contacting you because we believe you can make a valuable contribution, considering your position as…, to help us to identify such barriers.

Therefore, we are kindly requesting 15 minutes of your time to answer a brief set of questions to inform us about how you perceive the barriers for the sharing of microbial genetic data related to ownership of data and/or regulations that apply to them.

Prospectively, we will contact you via phone on the time schedule of…

More information about our study, and about COMPARE, can be found on the link http://www.compare-europe.eu/Project-organisation/Workpackages/Workpackage-12/Barriers-research. Please, do not hesitate to contact us in case you need any additional information, to reschedule or to cancel this appointment.

We are looking forward to learn about your perception(s) related to microbial genetic data sharing.

We thank you in advance.

Kind regards,

On behalf of the COMPARE WP12 team

Carolina Ribeiro and Martine van Roode.
