# Supplementary material for: How ownership rights over microorganisms affect infectious disease control and innovation: A root-cause analysis of barriers to data sharing as experienced by key stakeholders
Source: PLoS One. 2018 May 2;13(5):e0195885. doi: 10.1371/journal.pone.0195885 (PMC5931471; doi:10.1371/journal.pone.0195885)
Supplement: S2 File — (PDF) [file pone.0195885.s002.pdf]

S2. File. Description of the research on the COMPARE website.

COMPARE is a large project with the intention to speed up the detection of and response to disease outbreaks among humans and animals worldwide through the use of new genome technology. Our vision is to become the enabling analytical framework and globally linked data and information sharing platform for real-time analysis and interpretation of sequence-based pathogen data in combination with associated data (e.g., clinical, epidemiological data) in an integrated inter-sectorial, inter-disciplinary, international 'One Health' approach.

To be able to create such framework, barriers in data sharing need to be overcome. Therefore, the work package 12 from the COMPARE project was created to identify, clarify and, as far as feasible, develop practical solutions for barriers that hamper the timely and openly sharing of data through the COMPARE framework. Unless these barriers are better understood, solutions may remain deficient.

Correspondingly, ownership barriers for microbial genetic data sharing is defined, in this research, as instruments used to restrict data exchange, resulting from the underlying willingness and freedom (or not) to use, share, modify and profit from this data. Ownership, if appropriately understood, can allow decision makers to define when biological material should be shared on a non-exclusive basis and, conversely, when a restrictive licensing policy is justified.

That is why we want to know your perceptions in relation to ownership barriers for sharing microbial genetic data in such an open platform. In our interview we will ask you four questions to access your perspectives in relation to sharing microbial genetic data in such an open platform; which you consider to be barriers for this framework and how do you perceive these barriers to work (hamper the sharing).

We appreciate a lot your contribution and if wanted, feedback will be provided in relation to the identified barriers and their causes and consequences.
