# Supplementary material for: How ownership rights over microorganisms affect infectious disease control and innovation: A root-cause analysis of barriers to data sharing as experienced by key stakeholders
Source: PLoS One. 2018 May 2;13(5):e0195885. doi: 10.1371/journal.pone.0195885 (PMC5931471; doi:10.1371/journal.pone.0195885)
Supplement: S3 File — (PDF) [file pone.0195885.s003.pdf]

S3 File. Interview Guide.

Based on your experience / the experience of your institute as… we are contacting you to learn about your opinion on the sharing of microbial genetic data (MGD) in a publicly accessible international database. Before starting I would like to highlight that this interview will be performed according to the Chatham House rules, therefore the information provided will be kept anonymous. I also would like to ask your consent to record this interview for further analysis and use, anonymously, in future publications. Is there any point that you would like to clarify before starting the interview?

(1) Could you paint the position and involvement (on your daily work) of the sharing of MGD in a publicly accessible international database?

Further explanation:
confirm the stakeholder group (research institute, national surveillance center, supranational organization, or industry) and domain (human/animal/food) the key opinion leader (KOL) belongs to.

(2) Have you ever experienced or perceived any ownership barriers for the sharing of MGD in a publicly accessible international database?

Further explanation: ownership barriers are related to the underlying willingness and freedom (or not) to use, share, modify and profit from this data.

(3) So, you have mentioned the following barrier(s)… Why do you consider these barriers to hamper the sharing of MGD, can you explain to me how these barriers work?
(4) Would you like to complement your answer or add any information? Do you have any further question?

Thank you very much for your time. Your contribution is very important for our project. Could we contact you again in the future to provide our results with an overview of the identified barriers for further feedback? Could you recommend one or more persons who we could take up contact for our research?
